# Nuclear receptor-driven immunometabolic crosstalk: immune-centric pharmacology targeting the inflamed nexus

**DOI:** 10.3389/fcell.2025.1706384

**Published:** 2026-01-21

**Authors:** Yang Zhang, Zhenzhen Pei, Zhige Wen, Yupeng Chen, Haoshuo Wang, Weili Tan, Xin Chen, Jingbo Liu, Qing Ni

**Affiliations:** 1 Department of Endocrinology, Guang’anmen Hospital, China Academy of Chinese Medical Sciences, Beijing, China; 2 Hohhot Mongolian and Traditional Chinese Medicine Hospital, Hohhot, China

**Keywords:** immune-centric pharmacology, immunometabolic crosstalk, metaflammation, nuclear receptors, tissue-specific targeting, TREM2/PPARγ

## Abstract

Chronic low-grade inflammation (metaflammation) constitutes a shared pathological nexus in obesity, type 2 diabetes mellitus (T2DM), and non-alcoholic fatty liver disease (NAFLD). While current therapies primarily alleviate metabolic symptoms, they often neglect underlying immune dysregulation orchestrated by nuclear receptors (NRs). This review proposes immune-centric pharmacology, a paradigm directly targeting immunocytes (e.g., macrophages, T cells) through spatiotemporal modulation of NR-mediated immunometabolic crosstalk (e.g., PPARγ/δ, FXR, LXRs) to disrupt inflammation-perpetuating microenvironments. We emphasized NRs as main regulatory factors of immunophenotypic reprogramming, spanning the interactions of fat, liver, and intestinal immunity, and comprehensively summarized the multicellular structure of “inflamed nexus.” We gradually expand our discussion from the following three aspects: immune reprogramming targeting nr by novel biological agents (for example, TREM2 agonists activating PPARγ); advanced transmission systems enable tissue-specific NR regulation.; and the immunomodulatory mechanism of metabolic drugs utilizing the NR-immune axis. Current findings indicate that focused immunomodulation achieves via NR-guided immune-centric pharmacology represents a transformative framework for next-generation metabolic disease management, bridging pharmacological innovation with therapeutic translation.

## Highlights

- Propose immune-centric pharmacology and spatiotemporally target NRs to reprogram immunocytes.
- Reveal TREM2-PPARδ axis as a druggable switch.
- Develop tissue-specific NRs to overcome systemic toxicity.
- Repurpose metabolic drugs via NR-immunomodulation.

## 1 Introduction

In recent years, rising affluence and associated lifestyle shifts have precipitated a sharp global increase in the incidence of metabolic disorders. The escalating burden of obesity, type 2 diabetes mellitus (T2DM), and non-alcoholic fatty liver disease (NAFLD) continues to escalate, underscoring the limitations of conventional metabolic management (Misra et al., 2023). A paradigm-shifting insight over the past decades reveals that chronic low-grade inflammation (metaflammation) is not merely a consequence but a fundamental driver of these conditions, orchestrated by dysregulated nuclear receptor (NR) networks governing immunometabolic crosstalk (Prattichizzo et al., 2018; Guan et al., 2022). Immunometabolic crosstalk refers to the complex and bidirectional interaction between the immune system and cellular metabolism. It describes how metabolic states affect the function of immune cells and how immune responses reprogram metabolism throughout the body and within cells. Epidemiological evidence links systemic inflammation to metabolic dysregulation, while NR-dependent mediators (e.g., PPARγ-modulated adipokines, FXR-bile acid axis) and immune cell signatures (e.g., TREM2+ macrophages) underpin tissue-specific immunopathology (Guan et al., 2022; Pepys and Hirschfield, 2003; Chiavaroli et al., 2021; Tan et al., 2023). Metaflammation creates self-sustaining pathological niches in adipose tissue, liver, and gut, where maladaptive immune-parenchymal crosstalk directed by NRs like liver X receptors (LXRs) and glucocorticoid receptor (GR) fuels insulin resistance, lipotoxicity, and fibrosis (Gao et al., 2022).

Despite recognizing inflammation’s centrality, current first-line metabolic therapies often inadequately address core pathology due to neglect of NR-immunocyte circuits. Metformin shows minimal efficacy in reversing PPARγ-dependent macrophage polarization. Insulin may disrupt the transinhibition of glucocorticoid receptors, thereby exacerbating inflammation. Statins exhibit dissociation from immunomodulation due to incomplete LXR agonism in Kupffer cells (Foretz et al., 2023; Sakai et al., 2019; Kheirollahi et al., 2019; Ikonomopoulou et al., 2021; Endo-Umeda et al., 2018). Consequently, these agents manage symptoms without fundamentally disrupting NR-guided inflammatory circuits, perpetuating therapeutic escape.

Immune-Centric pharmacology is an emerging paradigm in pharmacological research and drug development. It attaches the immune system as main target and central regulator of therapeutic intervention. This critical therapeutic gap necessitates NR-driven immune-centric pharmacology, defined as “therapeutic strategies exploiting nuclear receptors to directly reprogram immunocytes and dismantle pathological inflammatory microenvironments, with consequent metabolic improvements emerging as secondary outcomes.” Unlike conventional drugs targeting metabolic pathways downstream of inflammation, immune-centric pharmacology proactively intervenes at the immunological epicenter through synergistic strategies. Firstly, the precision immune reprogramming via NR-targeted biologics. Secondly, the utilization of advanced spatiotemporal delivery systems enabling tissue-specific NR modulation (Panzitt and Wagner, 2021; Teodoro et al., 2011). Thirdly, the systematic re-evaluation of existing or emerging metabolic drugs for NR-dependent immunomodulatory mechanisms (Chen et al., 2017). These multifaceted strategies aim to disrupt pathological circuits at their source. This review synthesizes evidence for NR-guided immune-centric pharmacology as a transformative framework to achieve durable disease modification to alleviate or even dismantle the “inflamed nexus” at its source.

## 2 The NR-mediated immunometabloic and inflammatory circuits

### 2.1 Nuclear receptors as central hubs of metaflammation

Chronic metabolic inflammation, a hallmark of obesity-associated disorders, exhibits distinctly spatial organized dysregulation governed by nuclear receptors. This process strategically localizes within specialized tissue niches, notably visceral adipose depots, pancreatic islets, and hepatic sinusoids, in which nuclear receptors serve as master regulators of immunometabolic crosstalk. Within these distinct anatomical niches, nuclear receptors drive disease progression through three interconnected mechanisms. They orchestrate transcriptional reprogramming, interpret local metabolic signals, and guide lasting epigenomic changes. Dysregulated signaling among immune and stromal cells establishes self-sustaining inflammatory circuits through nuclear receptor-dependent mechanisms. This process, marked by aberrant cytokine secretion and ectopic lymphoid structures, perpetuates a chronic inflammatory state that drives both local tissue and systemic metabolic dysfunction. These hubs represent critical sites where immune-metabolic crosstalk fails, thereby sustaining a pathological feedback loop (Wu et al., 2023). Three key molecular mechanisms sustain this pathological state: PPARγ promoter hypermethylation perpetuates adipocyte insulin resistance; FXR SUMOylation impairs macrophage efferocytosis in NASH; and LXR phosphorylation (S196) sustains inflammasome activation (Deczkowska et al., 2020; Jaitin et al., 2019; Zhang et al., 2021; Zhang et al., 2023). These nuclear receptor-mediated mechanisms explain the limited efficacy of conventional anti-inflammatory therapies against metaflammation, highlighting NRs as promising therapeutic targets for sustained disease modification. Three key hubs illustrate this paradigm.

#### 2.1.1 Adipose tissue remodeling: PPARγ dysregulation fuels inflammatory circuits

Chronic energy surplus promotes white adipose tissue (WAT) expansion through adipocyte hypertrophy and hyperplasia, initiating tissue inflammation and dysfunction via suppression of PPARγ-regulated pathways. An early key event is hypoxia resulting from inadequate angiogenesis during adipose expansion. This hypoxic environment stabilizes HIF-1α which activates pro-inflammatory pathways and simultaneously suppresses PPARγ activity, which is a key regulator of macrophage polarization (Lee et al., 2013; Yamaguchi et al., 2017; Wen and Lee, 2015; Feng et al., 2020). Hypertrophied adipocytes release chemokines like CCL2/MCP-1 and saturated free fatty acids (e.g., palmitate) under metabolic stress. These signals activate TLR4 on resident adipose macrophages and establish a chemokine gradient that recruits circulating monocytes via CCR2-CCL2 pathways. Infiltrating monocytes differentiate into macrophages and form crown-like structures around damaged adipocytes, a process intensified by PPARγ deficiency (De Jesus Simao et al., 2024; Kanda et al., 2006).

While PPARγ in macrophages is well recognized for its role in governing polarization toward the anti-inflammatory M2 phenotype, the PPARγ signaling axis within adipocytes themselves also exerts a non-negligible impact on the local inflammatory microenvironment of WAT. Functional PPARγ in adipocytes is essential for maintaining adipocyte homeostasis, as it directly modulates the expression of genes involved in lipid storage and adipokine secretion. Under conditions of PPARγ impairment in adipocytes, there is a shift toward the secretion of pro-inflammatory adipokines alongside a reduction in anti-inflammatory adipokines such as adiponectin. Adiponectin acts through its receptors AdipoR1 and AdipoR2 on macrophages and endothelial cells to inhibit NF-κB-mediated pro-inflammatory cytokine production and enhance endothelial barrier function; the decline in adiponectin secretion caused by adipocyte PPARγ dysfunction thus removes a critical anti-inflammatory brake in WAT. Moreover, adipocyte PPARγ directly represses the transcription of genes encoding pro-inflammatory mediators (e.g., TNF-α and IL-6) in adipocytes themselves, and its downregulation leads to autonomous pro-inflammatory cytokine release from adipocytes that synergizes with macrophage-derived cytokines to amplify local inflammation. Additionally, adipocyte PPARγ deficiency disrupts lipid handling within WAT, promoting ectopic lipid deposition in adjacent stromal cells and further exacerbating the pro-inflammatory milieu by inducing lipotoxicity in non-adipocyte cell populations of the adipose tissue. PPARγ dysfunction amplifies cytokine production across WAT cellular compartments. Macrophages within CLS release excessive TNF-α and IL-6 which promote adipocyte insulin resistance through complementary mechanisms that TNF-α activates the JNK/TAB1 pathway inducing inhibitory serine phosphorylation of IRS-1, while IL-6 upregulates SOCS3, leading to ubiquitin-mediated degradation of insulin signaling components (Akieda-Asai et al., 2024). Together, these responses form a self-sustaining inflammatory loop. Subsequent insulin resistance and lipolysis increase saturated free fatty acids (FFAs) in the microenvironment, which reactivate macrophage inflammation through TLR4 signaling and enhance monocyte recruitment via endothelial activation (Hu et al., 2020). This vicious cycle promotes nuclear receptor-centered pathogenesis, featured by DNMT3a-mediated PPARγ promoter hypermethylation and exosomal miR-34a transfer from senescent adipocytes to macrophages, which collectively suppress PPARγ-driven M2 polarization (Pasupuleti et al., 2023; Yan et al., 2016; Kauppinen et al., 2013).

These changes persist long-term in adipose tissue, and PPARγ epigenetic silencing maintains inflammation and metabolic dysfunction even after weight loss highlighting its therapeutic relevance for sustained tissue remodeling. WAT-derived inflammatory signals and FFAs also systemically impair other metabolic organs, including the liver and pancreatic islets.

#### 2.1.2 TREM2-PPARδ crosstalk in pancreatic islets immunopathology

Triggering receptor expressed on myeloid cells 2 (TREM2) is an immunoreceptor tyrosine-based activation motif (ITAM)-coupled receptor predominantly expressed on macrophages and microglia. It is highly upregulated in lipid-associated macrophages in obese and insulin-resistant individuals. TREM2 binds anionic ligands, such as lipopolysaccharide, phospholipids, and amyloid proteins to initiate intracellular signaling (Zhong et al., 2024; Wang et al., 2020). Under physiological conditions, adipose-resident TREM2-expressing macrophages orchestrate lipid homeostasis through phagocytic clearance of lipotoxic material within CLS, reinforced by Wnt/β-catenin-mediated anti-inflammatory signaling that suppresses NF-κB activation and enhances cellular survival programs (Zheng et al., 2017; Kang et al., 2025). Wnt/β-catenin signaling enhances the phagocytic clearance of lipotoxic debris by promoting PPARδ-dependent fatty acid oxidation and inhibiting NF-κB. Under diabetic conditions islet amyloid polypeptide (IAPP) oligomers activate macrophage TREM2 signaling, initiating SYK (spleen tyrosine kinase)-dependent mitochondrial fission and oxidative stress (Wang et al., 2022a). In T2DM, preclinical evidence suggests that this sustained TREM2/SYK signaling may contribute to aberrant mitochondrial dynamics, particularly excessive fission mediated by Drp1(Dynamin-Related Protein 1), which is increasingly recognized as a feature of metabolic dysfunction. IAPP aggregated pathologically activate TREM2, triggering sustained SYK signaling that drives Drp1-mediated mitochondrial fragmentation. This disrupts mitochondrial function and impairs PPARδ activity, a key regulator of lipid catabolism. Consequent PPARδ dysfunction manifests as significantly impaired fatty acid oxidation and lipid utilization. This metabolic dysregulation creates a permissive environment for chronic, low-grade inflammation, characterized by inflammasome hyperactivity and elevated IL-1β levels. Intriguingly, reports also indicate the involvement of non-canonical inflammasome pathways contributing to IL-1β maturation in metabolic contexts, potentially acting independently or synergistically with canonical NLRP3 activation. Furthermore, within pancreatic β-cells, the convergence of metabolic stress, inflammation, and potentially disrupted nuclear receptor signaling (including PPARδ) is associated with epigenetic modifications leading to the silencing of insulin genes. While PPARδ has been shown to influence β-cell function and insulin secretion, the specific mechanistic link involving diminished PPARδ chromatin occupancy directly at insulin gene promoters remains an active area of investigation (Lee et al., 2023; Zheng et al., 2021; Zhang et al., 2025; Tanaka et al., 2022). Monocyte recruitment influences T2DM progression through PPARδ-dependent mechanisms. CCR2^+^ inflammatory monocytes are recruited via CCL2 signaling into pancreatic islets, where they amplify inflammation and suppress PPARδ activity in local β-cells and macrophages impairing lipid metabolism and anti-inflammatory responses. Conversely, CX3CR1^+^ patrolling monocytes, which normally support tissue resolution via PPARδ-mediated programs undergo functional impairment under diabetic conditions. Sustained TREM2 activation and metabolic stress disrupt PPARδ signaling in these cells, promoting a pro-inflammatory phenotype marked by p38 MAPK hyperactivation and reduced regulatory extracellular vesicle release. Thus, PPARδ dysfunction serves as a central node linking monocyte heterogeneity to islet inflammation and β-cell failure in T2DM (Colonna and Holtzman, 2025; Wang et al., 2021; Yesian et al., 2025). The dysfunctional TREM2-PPARδ axis drives immunometabolic dysregulation requiring stage-specific therapeutic strategies. In early disease, TREM2 agonism enhances amyloid clearance and promotes PPARδ-dependent anti-inflammatory polarization to restore metabolic homeostasis. In advanced stages with prevailing mitochondrial dysfunction and PPARδ impairment TREM2 antagonism is required to break the cycle of amyloid-induced stress and inflammation. Additional approaches include nanocarrier-delivered PPARδ agonists or miRNAs targeting the TREM2-PPARδ mitochondrial fission pathway. Such targeted strategies may overcome the limitations of conventional incretin therapies in islet amyloidosis enables immunomodulation of the TREM2-PPARδ switch (Fyfe et al., 2023; Pan et al., 2019; Wang et al., 2022b).

While the TREM2–PPARδ axis plays a central role in islet immunopathology associated with T2DM, its involvement and functional outcome vary considerably across other islet-inflammatory disorders.

In type 1 diabetes, where autoimmune-mediated β-cell destruction predominates, TREM2-expressing macrophages are implicated in the clearance of apoptotic β-cell debris. However, persistent immune activation may shift TREM2 signaling toward a pro-inflammatory phenotype, potentially dampening PPARδ-dependent resolution pathways. Experimental models indicate that PPARδ agonists can attenuate insulitis and preserve β-cell mass, but whether modulation of TREM2 enhances this effect remains to be established.

In metabolic-stress-induced islet inflammation, TREM2 may initially facilitate lipid clearance, whereas chronic activation leads to PPARδ suppression and inflammasome hyper-activation.

Emerging observations in gestational diabetes mellitus (GDM) suggest alterations in placental and islet macrophage profiles. Preliminary evidence points to a potential disruption of TREM2–PPARδ crosstalk that could participate in transient insulin resistance and β-cell dysfunction, although mechanistic studies in GDM are still scarce (Milan et al., 2025a).

The functional interaction between TREM2 and PPARδ indicates that their coordinated regulation may be more effective than single-target therapies, especially when phagocytosis clearance and metabolic reprogramming are impaired. Mechanistically, the activation of TREM2 enhances the phagocytic function and lipid uptake of macrophages, while PPARδ promotes fatty acid oxidation and mitochondrial integrity. However, the optimal timing, dosage, and disease context for such combination therapies still require systematic research.

#### 2.1.3 Liver: FXR/LXR-guided Kupffer cell dynamics in NAFLD/NASH

**FIGURE 1:**
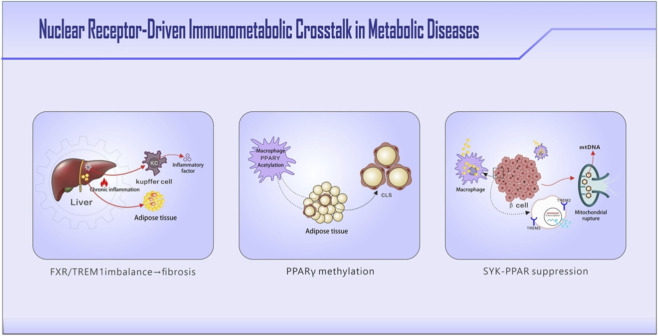
Overview of NR-driven immunometabolic circuits in adipose tissue, liver, and pancreatic islets. Key molecular players discussed throughout the manuscript are highlighted in bold. In the hepatic niche, lipid overload induces hepatocyte apoptosis, releasing sphingosine-1-phosphate. Impaired FXR/LXRα signaling reduces Kupffer cell efferocytosis, while endotoxin-activated TLR4 promotes ADAM-mediated TREM2 cleavage and TREM1-driven fibrogenesis. In the adipose tissue niche, hypertrophic adipocytes exhibit PPARγ promoter methylation, triggering CCL2-mediated monocyte recruitment. Crown-like structures of macrophages release TNF-α and IL-6, inducing adipocyte insulin resistance through IRS-1 serine phosphorylation and SOCS3 degradation. Free fatty acids reactivate TLR4 on macrophages, perpetuating a vicious cycle. In the islet niche, macrophage TREM2 hyperactivation by islet amyloid polypeptide oligomers drives SYK-dependent Drp1 phosphorylation, causing mitochondrial fission. This disrupts PPARδ nuclear translocation, impairing lipid oxidation and activating NLRP3 inflammasomes that suppress insulin genes in β-cells.

Kupffer cells (KCs), the liver-resident macrophages, orchestrate tissue homeostasis under physiological conditions through FXR and LXR-mediated transcriptional programs. During NAFLD progression, lipid-induced hepatocyte apoptosis releases sphingosine-1-phosphate (S1P), activating S1PR1 on recruited macrophages to upregulate TREM2 expression—a process potentiated by LXRα-dependent cholesterol efflux (Hou et al., 2020; Hu et al., 2019). TREM2+ macrophages execute nuclear receptor (NR)-coordinated metabolic functions: on one hand, they enhance efferocytosis of apoptotic cells via FXR, involving Rab5-GTPase-mediated vesicular trafficking; simultaneously, they drive fatty acid oxidation through LXRβ, relying on PPARδ co-activation; in addition, they suppress pro-inflammatory cytokine production via TREM2-TLR4 transrepression. Collectively, these functions attenuate hepatic steatosis (Wang et al., 2023). Conversely, gut-derived endotoxins activate TLR4-NF-κB signaling, which induces ADAM-mediated TREM2 cleavage while promoting TREM1 transcription. This pathogenic switch disrupts hepatic homeostasis through a cascade of events: by diminishing FXR-TREM2 coupling, it impairs lipid clearance; furthermore, through amplification of LXR-suppressed TREM1 cascades, it drives Kupffer cell depletion; and ultimately, via activation of stellate cells, it accelerates TGF-β-mediated fibrosis (Wang et al., 2023; Schlepckow et al., 2017; Calkin and Tontonoz, 2012). Therapeutically, NR targeted strategies correct this imbalance through coordinated interventions. Specifically, galectin-3 inhibitors preserve FXR activity by blocking TLR4 dimerization, thereby attenuating TREM1-driven inflammation; simultaneously, S1PR1 agonists enhance lipid metabolism through activation of the LXRα-TREM2 axis; while obeticholic acid (an FXR agonist) directly upregulates TREM2 expression to restore efferocytosis capacity. More importantly, combinatorial NR modulation—such as co-administration of an FXR agonist with an LXR partial agonist—synchronizes gut-liver immunity, thus reversing fibro-inflammatory cascades (Xie et al., 2023; Feng et al., 2018; Mackinnon et al., 2023; Wang et al., 2024; Potì et al., 2024) (Figure 1).

### 2.2 NRs as master signaling integrators in metaflammation

Targeting metabolic inflammation represents an effective therapeutic strategy for metabolic diseases. Metaflammation is orchestrated through evolutionarily conserved signaling hubs that transduce nutrient stress into immune dysregulation.

#### 2.2.1 NLRP3 inflammasome: transcriptional control by PPARγ and LXRs

Activation of NLRP3 leads to excessive secretion of inflammatory cytokines such as IL-1β, with three pivotal axes exhibiting broad therapeutic relevance (Olona et al., 2022). Metaflammation transduces nutrient stress into immune dysregulation through conserved hubs, with NLRP3 inflammasome activation serving as a pivotal axis. Wu et al. demonstrated that duodenojejunal bypass surgery in T2DM rats markedly improved glycemic and lipid profiles, upregulated glucokinase and GLUT2 expression, and significantly suppressed both mRNA and protein levels of the NLRP3 inflammasome (Wu et al., 2018). This further improves β-cell dysfunction and glucose tolerance; Further research has shown that knocking out the NLRP3 inflammasome in macrophages of T2DM rats can produce similar effects. Studies found that in knockout mice with genes such as NLRP3, apoptosis-associated speck-like protein (ASC) and caspase-1, palmitate activation of NLRP3 inflammasome would produce a large amount of active IL-1β and IL-18, thereby interfering with the insulin signaling pathway (Zheng et al., 2023; Zou et al., 2025). TXNIP, a protein associated with insulin resistance, contributes to NLRP3 inflammasome activation in hepatocytes. Silencing TXNIP completely blocks fructose-induced upregulation of NLRP3, ASC, and caspase-1 activation, but does not affect ROS generation. Conversely, inhibiting ROS production reduces TXNIP expression and decreases IL-1β and IL-18 levels (Zhang et al., 2024). NLRP3 inflammasome integrates diverse metabolic stressors, including cholesterol crystals, saturated fatty acids, and hyperglycemia induced TXNIP upregulation. PPARγ and LXRs regulate NLRP3 through distinct transcriptional mechanisms. PPARγ directly represses NLRP3 promoter activity and counters crystal- and lipid-induced oligomerization while LXRs inhibit TXNIP expression by downregulating METTL3, thereby reversing m^6^A-mediated TXNIP mRNA stabilization. Activation of the NLRP3-ASC-caspase1 cascade promotes IL-1β maturation which exacerbates systemic insulin resistance through JNK/IKKβ-mediated inhibitory phosphorylation of IRS-1. PPARγ agonists such as pioglitazone show superior efficacy over direct NLRP3 inhibitors by concurrently ameliorating insulin resistance via the PPARγ-IRS1 axis. Emerging METTL3 inhibitors (e.g., STM2457) suppress inflammasome activation and attenuate NAFLD-HCC progression via m^6^A-dependent silencing of oncogenes. Moreover, METTL3 ablation synergizes with immune checkpoint blockade by enhancing CD8^+^ T cell infiltration and antigen presentation, improving immunotherapy responses in NAFLD-HCC models and offering dual therapeutic benefits against metabolic inflammation (Milan et al., 2025b; Pan et al., 2023; Gao et al., 2023).

#### 2.2.2 cGAS-STING pathway: LXRs preserve mitochondrial integrity

The cGAS-STING pathway emerges as a critical inflammatory nexus, activated by cytosolic mitochondrial DNA (mtDNA) leakage resulting from oxidative damage. cGAS is mainly expressed in the cell nucleus and strictly bound to chromatin to prevent binding with nuclear DNA. Cytosolic mtDNA engages cGAS, synthesizing 2′,3′-cGAMP to stimulate STING protein. STING then recruits TANK-binding kinase 1 (TBK1), phosphorylating interferon regulatory factor 3 (IRF3) to drive type I interferon (IFN-α/β) production (Decout et al., 2021). Previous research showed that IFNs trigger adipocyte senescence via p38 mitogen-activated protein kinase (MAPK) activation, which is characterized by senescence-associated β-galactosidase (SA-β-gal) activity (Coppe et al., 2010). Cytosolic mtDNA leakage activates cGAS-STING signaling, driving IFN-α/β production. The specific mechanism by which liverX receptors regulate mtDNA integrity involves multiple approaches to enhance mitochondrial stability. LXR activation transcriptionally upregulates UCP2 (Uncoupling Protein 2), which dissipates the mitochondrial proton gradient to reduce membrane potential. This slight uncoupling reduces the driving force for superoxide generation on the electron transport chain, thereby limiting the production of ROS, which are the main cause of mtDNA damage. By alleviating oxidative stress, LXR signaling reduces DNA damage and mtDNA double-strand breaks, while stabilizing the inner mitochondrial membrane, preventing the pathological opening of permeable transition pores and subsequent rupture of the outer mitochondrial membrane, thereby allowing mtDNA to escape into the cytoplasm.

LXRs and PPARδ alleviate this cascade reaction through a dual complementary mechanism. LXRs targets upstream sources by maintaining mitochondrial integrity and preventing the leakage of initial mtDNA. PPARδ activation combats downstream aging responses by blocking p38 phosphorylation and aging-related β-galactosidase activity. This mechanism suggests that the effect of LXR/PPARδ may improve cellular senescence. When used in combination with anti-aging drugs, it is more effective in reversing adipose dysfunction than STING inhibitors alone, which only block one downstream pathway (Xu et al., 2015; Michelet et al., 2018; Pan et al., 2021; Islam et al., 2023).

#### 2.2.3 TREM2-SYK: a non-canonical PPARδ activation path

**FIGURE 2:**
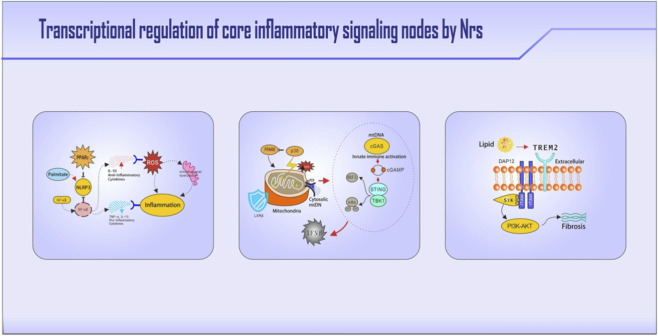
Nuclear receptor regulation of three pivotal immunometabolic pathways. Key molecules and regulatory nodes are highlighted in bold. The figure illustrates: (1) PPARγ suppresses NLRP3 inflammasome activation by blocking its transcription, while LXRs inhibit inflammasome priming via METTL3 downregulation; the NLRP3-ASC-caspase1 cascade drives IL-1β-mediated insulin resistance. (2) Leaked mitochondrial DNA activates cGAS-STING signaling, which is countered by LXRβ through mitochondrial stabilization (UCP2 induction) and PPARδ-mediated blockade of p38 MAPK-driven senescence. (3) TREM2-activated SYK signaling enables PPARδ nuclear translocation, cooperating with TFEB for lipid breakdown; under chronic lipid overload, SYK signaling is redirected to the PI3K-AKT-TGFβ profibrotic pathway.

The TREM2-SYK axis coordinates macrophage lipid metabolism through PPARδ-dependent reprogramming. When apolipoproteins or phospholipids such as ApoE bind to TREM2, they induce the phosphorylation of splenic tyrosine kinase (SYK), thereby initiating multiple downstream effector pathways (Wang et al., 2022a). Among them, SYK significantly enhances the efferocytosis ability of macrophages by activating the RAC1 protein to drive the recombination of the actin skeleton. At the same time, it promotes nuclear translocation of transcription factor EB (TFEB) and expands the lysosomal network to accelerate lipid hydrolysis. Concurrently, TREM2 ligation induces SYK phosphorylation, which directly triggers RAC1-mediated PPARδ nuclear translocation—bypassing classical ligand binding. PPARδ subsequently co-activates TFEB, synergistically expanding lysosomal networks for enhanced lipid hydrolysis. Chromatin immunoprecipitation sequencing (ChIP-seq) confirms PPARδ-TFEB co-occupancy on fatty acid oxidation gene promoters (Zheng et al., 2024; Jung et al., 2018). Under chronic lipid overload, SYK signaling is redirected toward the PI3K-AKT-TGFβ profibrotic pathway, thereby inactivating PPARδ. Specifically, when TREM2+ macrophages fail to resolve steatosis during early disease stages, persistent lipid excess shifts SYK signaling to phosphoinositide 3-kinase (PI3K)-AKT-mediated TGF-β secretion, accelerating fibrogenesis. Therapeutically, emerging agents like the TREM2 agonist AL-002 enhance lipid clearance, whereas in NASH models, the SYK inhibitor fostamatinib attenuates hepatic steatosis—highlighting context-dependent regulatory strategies (Wang et al., 2020; Long et al., 2024) (Figure 2).

#### 2.2.4 PPARγ and PPARδ in atherosclerosis immunometabolism regulation

Atherosclerosis, a lipid-driven chronic inflammatory disease of the arterial wall, exemplifies the central role of nuclear receptors in immunometabolic crosstalk. PPARγ and PPARα, two key members of the PPAR subfamily, are critical regulators that connect lipid homeostasis with vascular inflammation, thereby influencing disease progression and plaque stability.

PPARγ, expressed in vascular endothelial cells, macrophages, and smooth muscle cells, exerts atheroprotective effects through multiple mechanisms. In macrophages within atherosclerotic plaques, PPARγ activation promotes cholesterol efflux via transcriptional upregulation of the ABCA1 and ABCG1 transporters, reducing foam cell formation. Simultaneously, it suppresses pro-inflammatory gene expression by interfering with NF-κB and AP-1 signaling pathways, thereby attenuating vascular inflammation. Clinically, pioglitazone, a PPARγ agonist, has been shown to reduce carotid intima-media thickness and cardiovascular events in patients with T2DM, highlighting its translational relevance (Atas et al., 2025).

PPARα is mainly expressed in the liver and exists in vascular cells. It regulates systemic lipid metabolism by enhancing fatty acid β-oxidation and reducing circulating triglycerides. In the vascular environment, the activation of PPARα in endothelial cells inhibits the expression of adhesion molecules (such as VCAM-1), thereby reducing the recruitment of monocytes. In macrophages, it inhibits the activation of the NLRP3 inflammatory body and the secretion of IL-1β. Studies have shown that PPARα dysfunction is associated with an increased cardiovascular risk, and the clinically used PPARα agonist, the fibrates, can reduce cardiovascular events, especially in patients with atherosclerotic hyperlipidemia (Montaigne et al., 2021).

The PPARγ and PPARα signaling pathways have both distinct functions and overlapping functions in atherosclerosis. Their coordinated activation may bring about synergistic effects. PPARα can improve the drivers of dyslipidemia, while PPARγ can affect plaque inflammation and cholesterol deposition. However, their clinical applications also face some challenges, including fluid retention caused by PPARγ agonists and muscle disorders caused by fibrates. Emerging strategies, such as dual PPARα/γ agonists (e.g., saroglitazar), aim to utilize their complementary anti-atherosclerotic effects while reducing adverse consequences (Gawrieh et al., 2021). Future research should further analyze the cell type-specific roles of these receptors in the atherosclerotic microenvironment to achieve precise immune-metabolic regulation.

### 2.3 Bridging mechanisms and therapeutic strategies

Collectively, the NR-mediated circuits described above, spanning adipose PPARγ dysfunction, hepatic FXR/LXR imbalance, islet TREM2-PPARδ crosstalk, and integrative pathways such as NLRP3, cGAS-STING, and TREM2-SYK, which illustrate how nuclear receptors serve as central signaling hubs that coordinate immunometabolic responses in metabolic diseases. These receptors function as both key mediators of pathology and promising therapeutic targets, given their ability to sense metabolic stress and direct immune responses. Their dysregulation establishes self-sustaining inflammatory niches in tissues like fat, liver, and pancreas, driving disease progression. Therefore, precisely modulating NR activity to intercept these pathological circuits is a promising strategy for disease modification. Building on this mechanistic insight, the next-generation of immune-centric pharmacological approaches is emerging. In the following section, we explore these strategies, which include biologics, advanced delivery systems, and drug repurposing. All of them share the common goal of reprogramming NR-driven immunometabolism to resolve chronic inflammation and restore metabolic homeostasis.

## 3 Immune-centric pharmacological arsenal: NR-targeted strategies

### 3.1 Reprogramming immune phenotypes via NRs

#### 3.1.1 Biologics targeting macrophages reprogramming

Macrophages have significant phenotypic plasticity and can polarize into M1 or M2 types depending on the different stages of inflammation to adapt to their microenvironment. Macrophage phenotypic plasticity orchestrated by nuclear receptors enables microenvironment-adaptive polarization between pro-inflammatory (M1) and reparative (M2-like) states. Environmental adaptive changes prompt macrophages to undergo metabolic reprogramming, providing the cells with the necessary energy support, thereby effectively participating in anti-inflammatory responses, and regulating inflammatory processes. This plasticity hinges on NR-directed metabolic reprogramming wherein PPARγ, LXRs, and PPARδ shift glycolytic/oxidative phosphorylation balance to license anti-inflammatory responses. Novel biologic agents precisely target NR-immunometabolic checkpoints. The following are examples of the emerging strategies summarized.

##### 3.1.1.1 TREM2 agonist antibodies: PPARδ activation switches

TREM2 is expressed on lipid-associated macrophages (LAMs) in adipose tissue. The clinical-stage antibody AL002a, derived from the parental AL002 antibody, binds the extracellular IgV domain of TREM2. Knockout of TREM2 in mice leads to metabolic symptoms, including adipocyte hypertrophy, hypercholesterolemia, fat accumulation in the body and glucose intolerance (Reich et al., 2023). In Alzheimer’s disease models, TREM2 agonism by AL002 analogues induces SYK phosphorylation. PPARγ activation is associated with reparative functions in adipose tissue. TREM2 deficiency correlates with metabolic dysregulation including adipocyte hypertrophy, hypercholesterolemia, and glucose intolerance. While AL002a shows preclinical efficacy in ameliorating TREM2-related pathologies, the mechanistic link between SYK phosphorylation and PPARγ activation in AL002a-treated LAMs requires further experimental validation (Long et al., 2024; Xu et al., 2025).

##### 3.1.1.2 IL-4/IL-13 targeting adipose tissue macrophage immunopharmacology

IL-4 and IL-13 are classic cytokines associated with Th2-type immune responses. These cytokines activate the signal transducer and activator of transcription 6 (STAT6) to induce M2-like macrophage polarization, which might act as a key immunomodulatory axis for metabolic disease. Cold exposure induces IL-4-dependent adipose tissue browning, with IL-4/IL-13-deficient mice exhibiting impaired beige adipocyte formation. Receptor-interacting protein 140 (RIP140) is a coregulator of NRs that is highly expressed in adipose tissue. Research directly targeting RIP140 indicated that its knockout in macrophages increases the proportion of M2-type macrophages in the adipose tissue of obese mice by approximately threefold. Cardiac-specific RIP140 knockout has been shown to improve myocardial metabolism and prevent heart failure, but its role in adipose tissue macrophages remains unexplored. Based on correlative evidence, M2-like macrophages may promote beige adipogenesis, though direct mechanistic evidence is limited. The potential coordination between STAT6 and the nuclear receptor PPARγ in this process warrants further investigation. Notably, no existing studies demonstrate that M2 macrophages directly induce beige fat differentiation (Montaigne et al., 2021; Gawrieh et al., 2021; Reich et al., 2023). Therefore, the specific mechanisms by which M2-like macrophages regulate beige adipogenesis, particularly through potential STAT6-PPARγ crosstalk, warrant further investigation (Bernstein et al., 2023; Yamamoto et al., 2023; Ye et al., 2023).

##### 3.1.1.3 NR-Mediated immunomodulation of inflammasomes

Nuclear receptors emerge as master regulators of inflammasome pathways through immunometabolic mechanisms, offering novel therapeutic strategies for obesity-related pathologies. Specifically, the PPARγ signaling axis shows immunopharmacological relevance: PPARγ agonists (e.g., thiazolidinediones) suppress NLRP3 inflammasome assembly and IL-1β secretion in macrophages via transrepression of inflammatory genes, directly contributing to their efficacy in metabolic dysfunction-associated fatty liver disease (MAFLD). Notably, NR coregulators serve pivotal bridging functions, as exemplified by RIP140, a corepressor for PPARγ and other metabolic NRs highly expressed in adipose tissue macrophages. Macrophage-specific RIP140 knockout studies confirm that its ablation releases PPARγ suppression, enhancing M2 polarization, attenuating NLRP3/IL-1β-driven adipose inflammation, and improving insulin sensitivity in obese mice, thereby positioning RIP140 as a druggable interface linking NR signaling to immunometabolism (Yamamoto et al., 2023). Expanding this paradigm to NR-inflammasome crosstalk, although direct NLRP3 inhibitors like MCC900 demonstrate efficacy in MAFLD (Jiang et al., 2017), their actions require functional NR pathways: MCC950s anti-fibrotic effects depend on intact PPARγ signaling in myeloid cells, while PPARγ agonists potentiate MCC950-mediated suppression of cholesterol crystal-induced NLRP3 activation. This synergy underscores combinatorial targeting of NR-inflammasome axes as a promising therapeutic approach. Furthermore, liver X receptor (LXR) agonists (e.g., GW3965) transcriptionally repress NLRP3 expression and promote cholesterol efflux, establishing direct NR-mediated immunopharmacological control over inflammasomes in NAFLD models. Collectively, targeting NRs (PPARγ, LXR) and their coregulators (RIP140) enables precise immunomodulation of NLRP3-driven metabolic inflammation. Future research should prioritize elucidating NR-STAT6/PPARγ coordination in macrophage polarization, developing dual-targeting NR/NLRP3 modulators with optimized drug properties, and exploring extracellular vesicle-mediated NR immunometabolic pathways.

#### 3.1.2 Small molecules modulating NR-immunometabolism

Nuclear receptor-targeted small molecules reprogram immunometabolic circuits by leveraging the structural plasticity of ligand-binding domains (LBDs). Nuclear receptor ligands achieve high-affinity binding to specific pockets within the ligand-binding domains, such as PPARγ′s hydrophobic cavity or FXR’s bile acid recognition site. These interactions trigger structural rearrangements that control how transcriptional coregulators are recruited. This includes displacement of corepressors (e.g., NCOR1, NCOR2/SMRT) and stabilization of coactivators (e.g., PPARGC1A/PGC-1α, MED1/DRIP205), driving an allosterically regulated coregulator exchange (Ritter et al., 2021; Yue et al., 2011). Consequently, NR ligands exert tissue and cell type-specific control over macrophage polarization states and T-cell differentiation, directly coupling metabolic sensing to immunological responses. Two prototypical agents exemplify this paradigm-shifting strategy.

##### 3.1.2.1 PPARγ agonists (Thiazolidinediones): reprogramming adipose immunity

Thiazolidinediones (TZDs) are ligands of the PPARγ receptor, by binding PPARγ’s ligand-binding domain, can induce allosteric shifts that recruit coactivators, orchestrating immunometabolic reprogramming with cell type-specific outcomes. Critically, TZDs suppress adipose inflammation through PPARγ-mediated interference with NF-κB signaling and thus reducing inflammasome activity. Conversely, environmental toxicants like per- and polyfluoroalkyl substances (PFAS) exploit the homologous PPARα-ACOX1 axis to disrupt hepatic lipid homeostasis, inducing oxidative stress and steatosis through ACOX1 overactivation. This contrast underscores the duality of NR signaling: In adipocytes, TZDs upregulate lipid storage genes (CD36/FABP4), driving ectopic lipid accumulation. In macrophages, PPARγ activation enhances oxidative metabolism (e.g., ACOX1-mediated peroxisomal β-oxidation) to promote M2 polarization counteracting the pathological ACOX1 hyperactivity seen in PFAS-induced metabolic disorders. In hepatocytes, PFAS hijack PPARα to hyperactivate acox1-diverging from TZD-PPARγ’s physiological ACOX1 modulation (Cariou et al., 2012; Lee et al., 2021; Zhou et al., 2025). Such NR pathway duality extends to T-cell compartments, where PPARγ activation expands immunoregulatory subsets while impairing cytotoxic responses a functional dichotomy that reflects context-dependent nuclear receptor pharmacology in metabolic disease pathogenesis. To resolve this paradox, next-generation tissue-selective PPARγ modulators such as INT131 (Motani et al., 2009) dissociate metabolic benefits from pro-inflammatory lipogenesis through two complementary strategies: sparing SREBP1c transactivation via biased ligand-binding domain conformations and preserving regulatory T-cell expansion by maintaining FOXP3-coactivator retention complexes, thereby exemplifying precision immunopharmacology for next-generation metabolic therapy.

##### 3.1.2.2 FXR agonists: orchestrating gut-liver immunity

Obeticholic acid (OCA) is a farnesoid X receptor (FXR) agonist and a novel derivative of chenodeoxycholic acid (CDCA), one of the primary bile acids in humans. Among them, FXR is a key regulatory molecule for bile acids, inflammatory responses, fibrosis, and metabolic pathways. Obeticholic acid (OCA) activates systemic FXR with intestinal predominance to reshape gut-liver immunity. By binding FXR in ileal enterocytes, OCA induces fibroblast growth factor 19 (FGF19) secretion. This hormone suppresses hepatic bile acid synthesis via CYP7A1 downregulation, reducing pools of toxic bile acids that activate pro-inflammatory signaling through macrophage TGR5 and TLR4. Concurrently, intestinal FXR signaling induces Small Heterodimer Partner (SHP) to repress retinoic acid receptor-related orphan receptor γt (RORγt) activity in lamina propria mononuclear cells thereby inhibiting IL-23-driven expansion of Th17 cells—a key pathway in metabolic inflammation. In the regenerate phase 3 trial for NASH, OCA exhibited targeted immunometabolic actions. After 18 months of treatment with 25 mg/day OCA, significantly more patients achieved fibrosis improvement without disease progression compared to placebo (23% vs. 12%). This antifibrotic effect paralleled reductions in hepatic inflammatory activity. Pruritus occurred dose-dependently in 33% of OCA-treated patients, consistent with systemic FXR activation. Concurrently, OCA enhanced intestinal barrier integrity, demonstrated through decreased fecal zonulin and elevated mucosal tight junction protein expression (Younossi et al., 2019). Emerging gut restricted FXR agonists may offer a promising approach to circumvent these limitations. Preclinical and early clinical evidence suggests that compounds such as cilofexor could selectively activate intestinal FXR while minimizing systemic exposure (Patel et al., 2020; Fuchs et al., 2023). This localized targeting might preserve beneficial FXR-IL-10 axis signaling in gut macrophages, potentially enhancing antimicrobial peptide secretion, and reducing endotoxemia (Fu et al., 2022). Such tissue-specific strategies appear to maintain metabolic efficacy while likely mitigating pruritus risk—positioning them as investigational approaches for restoring gut-liver immune homeostasis (Clifford et al., 2021).

### 3.2 Advanced delivery systems: spatiotemporal precision for NR modulation

#### 3.2.1 Liver-targeted LXR nanocarriers overcome systemic toxicity barriers

Galactose-functionalized polymeric nanoparticles (e.g., biocompatible PLGA-PEG) deliver agonists like T0901317 specifically to hepatocytes through asialoglycoprotein receptor (ASGPR)-mediated uptake. This hepatocyte-restricted targeting prevents LXRα activation in peripheral tissues avoiding hypertriglyceridemia, a major limitation of systemic LXR therapy (Wang et al., 2022b; Sharma et al., 2021; Rudalska et al., 2021). Within hepatocytes, LXR agonists upregulate ABCA1 transporters to enhance cholesterol efflux (Chawla et al., 2001). This reduces intracellular lipid accumulation in preclinical NASH models, potentially limiting foam cell formation in hepatic macrophages. Co-delivered Kupffer cell-targeted siRNA suppresses TLR4/NF-κB pathways attenuating pro-inflammatory cytokine production. This liver-targeted approach holds relevance for NASH, a disease characterized by the triumvirate of steatosis, inflammation, and fibrosis. By compartmentalizing LXR activation to hepatocytes, the strategy directly addresses hepatic lipid accumulation, while the co-targeting of Kupffer cells mitigates the inflammatory response that fuels disease progression. More importantly, by suppressing Kupffer cell activation, this approach may indirectly attenuate hepatic stellate cell activation and subsequent fibrogenesis, thereby intervening at multiple nodal points in the NASH pathological cascade (Yang et al., 2021). This spatially stratified delivery logic exemplifies how advanced nanocarriers can be rationally designed to dissect and target the multicellular pathology of metabolic liver disease, moving beyond mere tissue targeting to cellular niche-specific intervention.

#### 3.2.2 Spatially defined immunometabolic reprogramming via adipose-targeted PPARγ delivery

Hyaluronic acid-conjugated liposomes loaded with rosiglitazone preferentially accumulate in visceral adipose tissue through CD44 receptor-mediated targeting (Hayward et al., 2016). This approach significantly enhances M2 macrophage polarization within CLS by sustaining local PPARγ activation, while minimizing PPARγ engagement in bone marrow. Such spatial selectivity mitigates the risk of osteoporosis, a recognized limitation of systemic thiazolidinediones. The pathological rationale for adipose-specific targeting extends beyond avoiding side effects; it directly addresses the systemic metabolic crosstalk central to T2DM. Visceral adipose tissue is a primary source of pro-inflammatory cytokines (e.g., TNF-α, IL-6) and adipokines that contribute to peripheral insulin resistance and pancreatic β-cell dysfunction. By precisely reprogramming ATMs within the visceral depot, this strategy aims to dismantle the inflamed adipose-liver-islet axis. Reducing adipose-derived inflammatory signals can alleviate the lipotoxic and inflammatory stress on pancreatic islets, potentially preserving β-cell function. Therefore, adipose-targeted PPARγ delivery is not merely a safety refinement but a pathology-aware therapeutic strategy designed to intercept the adipose-centric inflammatory drivers of systemic metabolic dysfunction.

### 3.3 Repurposed metabolic drugs: releasing NR immunomodulatory potential

#### 3.3.1 Metformin reprograms immunometabolism through AMPK-PPARγ crosstalk

Metformin exerts significant anti-inflammatory and metabolic effects, primarily mediated by activating AMP-activated protein kinase (AMPK) within ATMs. This AMPK activation initiates signaling cascades that culminate in the activation of p38 mitogen-activated protein kinase (p38 MAPK) (Yee et al., 2012). Activated p38 MAPK subsequently induces the phosphorylation of PPARγ. PPARγ phosphorylation potently enhances its suppressive capacity, enabling it to robustly suppress the assembly and activation of the NLRP3 inflammasome, thereby dampening macrophage pro-inflammatory responses (Jafarzadeh et al., 2025). Simultaneously, phosphorylated PPARγ potentiates the expression of genes critical for fatty acid oxidation including carnitine palmitoyl transferase 1A (CPT1A) and pyruvate dehydrogenase kinase 4 (PDK4) (Niu et al., 2023). Collectively, this suppression of inflammasome activity and promotion of fatty acid oxidation, mediated by the AMPK/p38 MAPK/PPARγ axis synergizes to drive a phenotypic shift in ATMs. This shift transitions macrophages from a pro-inflammatory state towards a tissue-reparative, anti-inflammatory state. Clinically, metformin treatment T2D patients is associated with beneficial adipose tissue niche remodeling, evidenced by a reduction in CLS–histopathological markers of inflammation where macrophages engulf dead adipocytes (Zhou et al., 2018). The observed reduction in CLS provides clinical evidence consistent with the proposed NR-mediated, highlighting metformin’s benefits extending beyond glycemic control.

#### 3.3.2 Statins paradoxically suppress LXR-ABCA1 cholesterol efflux in immune cells

**FIGURE 3:**
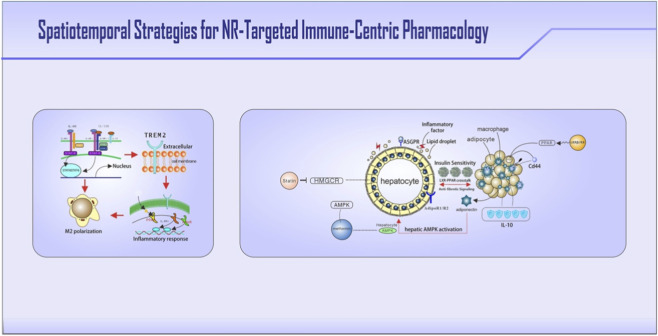
Three strategic approaches to modulate NR-immune circuits in metabolic diseases. Core therapeutic agents, targets, and pathways are highlighted in bold. The figure illustrates: (1) Biologics: e.g., TREM2 agonistic antibodies activate PPARγ in macrophages to resolve inflammation; targeting the IL-4/IL-13 axis promotes tissue-reparative macrophage polarization. (2) Advanced delivery systems: e.g., galactose-coated nanoparticles enable liver-specific LXR agonist delivery via ASGPR-mediated targeting; hyaluronic acid-conjugated liposomes direct PPARγ agonists to adipose tissue through CD44 receptor engagement. (3) Repurposed drugs: e.g., metformin activates the AMPK-p38-PPARγ axis to suppress inflammasome activation; combining statins with LXRβ agonists restores macrophage cholesterol efflux via ABCA1.

Statins, the cornerstone therapy for lowering low-density lipoprotein cholesterol (LDL-C), function by potently inhibiting the enzyme HMG-CoA reductase. This inhibition effectively blocks the mevalonate pathway, the primary route for endogenous cholesterol biosynthesis, leading to the desired reduction in circulating LDL-C. However, this inhibition has a significant downstream metabolic consequence: it concurrently depletes cellular pools of isoprenoid intermediates. A key affected metabolite is geranylgeranyl pyrophosphate (GGPP). GGPP serves as a crucial metabolic precursor for the endogenous synthesis of oxysterols, which act as ligands that activate LXRα. Statin-induced GGPP depletion reduces LXRα activation within macrophages reduces LXRα activity directly impairs its ability to drive the transcription of the ATP-binding cassette transporter A1 (ABCA1) (Fan et al., 2012; Yan et al., 2018; Ito et al., 2015). Excess intracellular cholesterol accumulation is a well-established trigger for macrophage activation, leading to the amplified secretion of pro-inflammatory cytokines via mechanisms such as NLRP3 inflammasome activation. To overcome this limitation inherent to statin monotherapy, next-generation adjunctive therapies are being developed. LXRβ-selective agonists, such as BMS-779788 represent a promising class (Kirchgessner et al., 2015). These agents are designed to potently activate LXRβ the isoform predominantly expressed in macrophages and critical for regulating ABCA1.Crucially, LXRβ-selective agonists demonstrate minimal activity on LXRα the isoform highly expressed in the liver whose activation drives lipogenic gene expression (e.g., SREBP-1c) and can cause hepatic steatosis (Rong et al., 2017). Therefore, BMS-779788 and similar compounds can effectively restore ABCA1 expression and cholesterol efflux flux in macrophages without inducing the undesirable hepatic lipogenesis associated with non-selective LXR agonists. This strategy exemplifies how precision co-targeting of NRs and specifically, inhibiting HMG-CoA reductase, aiming to enhance their overall benefit-risk profile by mitigating pro-inflammatory side effects while maintaining potent LDL-lowering (Table 1) (Figure 3).

**TABLE 1:** Clinical-translational landscape of NR-Targeted metabolic immunomodulators.

Target	Agent type	Example compound	Efficacy highlights	Key Limitations
PPARγ	Tissue-selective agonists	INT131	Dissociated lipid production/insulin sensitization	Risk of bone marrow suppression
FXR	Intestinal restrictive agonists	Cilofexor	Improve barrier function and there is no generalized itching	The anti-fibrotic efficacy of monotherapy is insufficient
TREM2	Agonist antibody	AL002a	Restore lipid phagocytosis in macrophages	Dose-dependent eosinophilia

### 3.4 Therapeutic challenges: navigating NR immunopharmacology complexities

Achieving tissue-selective NR modulation remains a paramount yet elusive goal. Systemic glucocorticoid receptor (GR) agonism exemplifies this challenge: while conferring anti-inflammatory benefits in adipose niches, it concurrently induces osteoporosis and muscle wasting via off-target transcriptional activation in bone and skeletal muscle. These adverse effects arise from evolutionarily conserved DNA-binding domain (DBD) homology across the NR superfamily, which enables nonspecific chromatin binding beyond therapeutically intended genomic sites (Escoter-Torres et al., 2020). Heterodimer partner selection introduces additional complexity for precision targeting. The obligate RXR heterodimerization of PPARγ, LXR and FXR exhibits tissue-specific dimerization kinetics - hepatic LXRα preferentially pairs with RXRα whereas intestinal FXR favors RXRγ (Le Maire et al., 2009). Tissue-selective RXR heterodimerization is governed by conformational dynamics. Pan-RXR agonists disrupt tissue-selective dimerization, driving lipogenesis via hepatic SREBP1c upregulation while impairing FXR-RXRγ-mediated cytoprotective functions in intestine (Nahoum et al., 2007; Adouvi et al., 2023).

**FIGURE 4:**
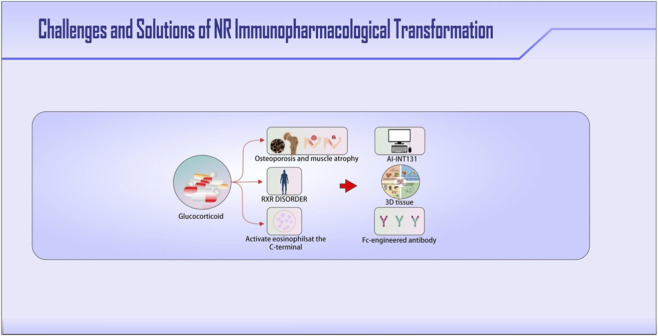
Key translational challenges and emerging solutions in NR-targeted immunopharmacology. Central challenges and strategic solutions are highlighted in bold. The diagram outlines major hurdles: (1) achieving tissue-selective effects is hindered by conserved NR structures leading to off-target impacts (e.g., bone loss via GR activation), and by complex, tissue-specific RXR heterodimer partnerships (2) immune-modulating biologics (e.g., TREM2 antibodies); can induce off-tissue immune reactions (e.g., eosinophilia). Proposed solutions include: designing biased ligands using AI-guided drug design (e.g., INT131), profiling tissue-specific NR-RXR dimerization in organoid models, and employing antibody engineering strategies to restrict cell-type-specific delivery and minimize side effects.

Clinical translation encounters immunotoxicity challenges, exemplified by TREM2 agonists such as AL002. Despite demonstrating efficacy in ameliorating metabolically driven inflammation, dose-dependent peripheral eosinophilia emerges as a dose-limiting adverse effect. This off-tissue effect stems from AL002s Fc domain engaging macrophage FcγRIIA triggering GM-CSF-mediated bone marrow eosinophil mobilization. Such observations underscore the necessity for cell-type-specific delivery strategies in immune-modulating therapeutics. Collectively, these challenges necessitate next-generation precision solutions: artificial intelligence-guided NR isoform design, organoid-based dimerization profiling, and antibody engineering for immune cell-restricted delivery—paving the path toward clinically viable NR immunopharmacology (Figure 4).

## 4 Conclusion and perspectives

The paradigm of nuclear receptor (NR)-driven immunometabolic crosstalk redefines therapeutic approaches for obesity, type 2 diabetes, and non-alcoholic fatty liver disease by positioning chronic inflammation as a druggable nexus. While conventional metabolic therapies alleviate symptoms, they fail to dismantle self-perpetuating inflammatory circuits orchestrated by dysregulated NR networks in adipose, hepatic, and islet microenvironments. Immune-centric pharmacology targeting NR-immunocyte axes with spatiotemporal precision holds transformative potential for durable disease modification. However, its efficacy hinges critically on tissue- and cell type-specific NR modulation, as systemic NR activation faces significant limitations: off-target effects, functional duality, and heterodimer complexity. These challenges underscore the necessity for NR-spatiotemporal precision to avoid paradoxical immunometabolic outcomes.

To overcome current limitations, integrated strategies focus on three pillars. Firstly, tissue-specific ligand design leverages compartmentalized targeting: hepatocyte-directed LXR agonists promote cholesterol efflux without hypertriglyceridemia; visceral adipose-targeted PPARγ delivery polarizes CLS macrophages without bone marrow toxicity; and gut-restricted FXR agonists preserve intestinal barrier integrity while mitigating systemic pruritus. Secondly, AI-driven NR heterodimer regulation employs machine learning to predict RXR isoform binding landscapes, organoid-based screening to profile NR heterodimers in diseased niches, and computational modeling of ligand-binding domain dynamics to engineer biased ligands. Thirdly, combination with immune checkpoint inhibitors exploits NR regulation of immunogenic cell death and T-cell exhaustion: PPARγ agonists synergize with PD-1 blockade to reverse CD8^+^ T-cell dysfunction in adipose/NAFLD microenvironments and enhance antitumor immunity, while LXRβ agonism remodels immunosuppressive niches by restoring macrophage cholesterol efflux.

Bridging innovation to clinical practice requires a chronologically stratified approach. In early disease, TREM2 agonism enhances amyloid/lipid clearance and PPARδ-dependent macrophage reprogramming. For advanced fibrosis, SYK inhibition interrupts NR-dysregulated profibrotic loops. Concurrently, repurposed drugs demonstrate NR-immunomodulatory potential: metformin modulates the AMPK/p38/PPARγ axis to suppress NLRP3 inflammasome activation, while statin-LXRβ combinations counteract ABCA1 suppression. Accelerating translation necessitates convergence of spatial NR pharmacology, computational biology, and immunometabolic circuit mapping to optimize targeted interventions across disease stages.

Expanding the indications of nuclear receptor ligands that are already in clinical use or advanced development represents a practical route to accelerate translation. This strategy can be illustrated with several existing facts. For instance, FXR agonists such as obeticholic acid, initially approved for primary biliary cholangitis, are now being actively investigated for their ability to target metabolic inflammation in conditions like NASH. Similarly, the well-known PPARγ modulator pioglitazone is being re-evaluated not only for glycemic control but also for its direct anti-inflammatory effects on diabetic cardiovascular and renal systems. Furthermore, PPARα agonists of the fibrate class may offer benefits in mitigating vascular inflammation beyond their classical lipid-lowering role.

Redirecting these therapies toward immune-inflammatory indications will depend on several factors. These include reassessing optimal dosing, understanding tissue-specific exposure, and confirming long-term safety in new patient groups. Overall, this repurposing approach, grounded in the principles of immune-centric pharmacology, offers a viable strategy to rapidly deliver mechanism-based treatments for the inflammatory underpinnings of metabolic diseases.

Key research priorities include deciphering NR-STAT6/PPARγ crosstalk in macrophage-driven beige adipogenesis, developing senolytics targeting NR-associated inflammation (Guo et al., 2024), and validating extracellular vesicle-mediated NR signaling as novel druggable targets. In conclusion, immune-centric pharmacology anchored in NR-spatiotemporal precision heralds a new era of mechanism-driven metabolic therapeutics. By dismantling the inflamed nexus at its source, this paradigm integrates molecular discovery with transformative clinical outcomes, offering sustainable solutions for complex metabolic diseases.

## Glossary

ABCA1: ATP-binding Cassette Transporter A1
ABCG1: ATP-binding Cassette subfamily G member 1
ADAM: A Disintegrin and Metalloproteinase
AKT: Protein Kinase B
AMPK: AMP-activated Protein Kinase
ASC: Apoptosis-associated Speck-like protein containing a CARD
ASGPR: Asialoglycoprotein Receptor
ATMs: Adipose Tissue Macrophages
CCL2: Chemokine (C-C motif) Ligand 2 (also known as MCP-1)
CCR2: C-C Chemokine Receptor Type 2
CD36: Cluster of Differentiation 36
CLS: Crown-like Structures
CPT1A: Carnitine Palmitoyl Transferase 1A
CRP: C-reactive Protein
CX3CR1: C-X3-C Motif Chemokine Receptor 1
CYP7A1: Cholesterol 7 alpha-hydroxylase
cGAS: cyclic GMP-AMP Synthase
DBD: DNA-binding Domain
Drp1: Dynamin-Related Protein 1
FABP4: Fatty Acid Binding Protein 4
FGF19: Fibroblast Growth Factor 19
FXR: Farnesoid X Receptor
GGPP: Geranylgeranyl Pyrophosphate
GM-CSF: Granulocyte-Macrophage Colony-Stimulating Factor
GR: Glucocorticoid Receptor
HIF-1α: Hypoxia-Inducible Factor 1-alpha
HMG-CoA: 3-Hydroxy-3-Methylglutaryl-CoA
IAPP: Islet Amyloid Polypeptide
IFN: Interferon
IL-1β: Interleukin-1 beta
IL-4: Interleukin-4
IL-6: Interleukin-6
IL-10: Interleukin-10
IL-13: Interleukin-13
IL-18: Interleukin-18
IL-23: Interleukin-23
IRS-1: Insulin Receptor Substrate 1
ITAM: Immunoreceptor Tyrosine-based Activation Motif
JNK: c-Jun N-terminal Kinase
KCs: Kupffer Cells
LAMs: Lipid-associated Macrophages
LBD: Ligand-binding Domain
LDL-C: Low-Density Lipoprotein Cholesterol
LXR: Liver X Receptor
LXRs: Liver X Receptors
MAPK: Mitogen-Activated Protein Kinase
MCP-1: Monocyte Chemoattractant Protein-1 (same as CCL2)
METTL3: Methyltransferase-like 3mmc1
mtDNA: Mitochondrial DNA
NAFLD: Non-alcoholic Fatty Liver Disease
NASH: Non-alcoholic Steatohepatitis
NEK7: NIMA-related Kinase 7
NF-κB: Nuclear Factor kappa-light-chain-enhancer of activated B cells
NLRP3: NOD-like receptor family, pyrin domain containing 3
NR: Nuclear Receptor
NRs: Nuclear Receptors|
OCA: Obeticholic Acid
p38 MAPK: p38 Mitogen-Activated Protein Kinase
PD-1: Programmed Death-1mmc2
PDK4: Pyruvate Dehydrogenase Kinase 4
PI3K: Phosphoinositide 3-Kinase
PLGA-PEG: Poly (lactic-co-glycolic acid)-Polyethylene Glycol
PPAR: Peroxisome Proliferator-Activated Receptor
PPARγ: Peroxisome Proliferator-Activated Receptor gamma
PPARδ: Peroxisome Proliferator-Activated Receptor delta
RAC1: Ras-related C3 botulinum toxin substrate 1
RIP140: Receptor-interacting protein 140
RORγt: Retinoic acid receptor-related orphan receptor gamma t
ROS: Reactive Oxygen Species
RXR: Retinoid X Receptor
SA-β-gal: Senescence-associated β-galactosidase
SHP: Small Heterodimer Partner
SOCS3: Suppressor of Cytokine Signaling 3
SREBP1c: Sterol Regulatory Element-Binding Transcription Factor 1c
STAT6: Signal Transducer and Activator of Transcription 6
STING: Stimulator of Interferon Genes
SYK: Spleen Tyrosine Kinase
TBK1: TANK-binding Kinase 1
TGF-β: Transforming Growth Factor beta
Th17: T helper 17 cells
TLR4: Toll-like Receptor 4
TNF-α: Tumor Necrosis Factor alpha
TREM1: Triggering Receptor Expressed on Myeloid cells 1
TREM2: Triggering Receptor Expressed on Myeloid cells 2
T2DM: Type 2 Diabetes Mellitus
TXNIP: Thioredoxin Interacting Protein
UCP2: Uncoupling Protein 2
WAT: White Adipose Tissue
Wnt: Wingless-type MMTV integration site family

## References

[B1] Adouvi G. Isigkeit L. López-García Ú. Chaikuad A. Marschner J. A. Schubert-Zsilavecz M. (2023). Rational design of a new RXR agonist scaffold enabling single-subtype preference for RXRα, RXRβ, and RXRγ. J. Med. Chem. 66 (1), 333–344. 10.1021/acs.jmedchem.2c01266 36533416

[B2] Akieda-Asai S. Ma H. Han W. Nagata J. Yamaguchi F. Date Y. (2024). Mechanism of muscle atrophy in a normal-weight rat model of type 2 diabetes established by using a soft-pellet diet. Sci. Rep. 14 (1), 7670. 10.1038/s41598-024-57727-2 38561446 PMC10984920

[B3] Atas E. Berchtold K. Schlederer M. Prodinger S. Sternberg F. Pucci P. (2025). The anti-diabetic PPARγ agonist pioglitazone inhibits cell proliferation and induces metabolic reprogramming in prostate cancer. Mol. Cancer 24 (1), 134. 10.1186/s12943-025-02320-y 40320521 PMC12051277

[B4] Bernstein Z. J. Shenoy A. Chen A. Heller N. M. Spangler J. B. (2023). Engineering the IL-4/IL-13 axis for targeted immune modulation. Immunol. Rev. 320 (1), 29–57. 10.1111/imr.13230 37283511

[B5] Calkin A. C. Tontonoz P. (2012). Transcriptional integration of metabolism by the nuclear sterol-activated receptors LXR and FXR. Nat. Rev. Mol. Cell. Biol. 13 (4), 213–224. 10.1038/nrm3312 22414897 PMC3597092

[B6] Cariou B. Charbonnel B. Staels B. (2012). Thiazolidinediones and PPARγ agonists: time for a reassessment. Trends Endocrinol. Metab. 23 (5), 205–215. 10.1016/j.tem.2012.03.001 22513163

[B7] Chawla A. Boisvert W. A. Lee C. H. Laffitte B. A. Barak Y. Joseph S. B. (2001). A PPAR gamma-LXR-ABCA1 pathway in macrophages is involved in cholesterol efflux and atherogenesis. Mol. Cell. 7 (1), 161–171. 10.1016/s1097-2765(01)00164-2 11172721

[B8] Chen S. C. Brooks R. Houskeeper J. Bremner S. K. Dunlop J. Viollet B. (2017). Metformin suppresses adipogenesis through both AMP-Activated protein kinase (AMPK)-Dependent and AMPK-Independent mechanisms. Mol. Cell. Endocrinol. 440, 57–68. 10.1016/j.mce.2016.11.011 27856330 PMC5228588

[B9] Chiavaroli L. Lee D. Ahmed A. Cheung A. Khan T. A. Blanco S. (2021). Effect of low glycaemic index or load dietary patterns on glycaemic control and cardiometabolic risk factors in diabetes: systematic review and meta-analysis of randomised controlled trials. BMJ 374, n1651. 10.1136/bmj.n1651 34348965 PMC8336013

[B10] Clifford B. L. Sedgeman L. R. Williams K. J. Morand P. Cheng A. Jarrett K. E. (2021). FXR activation protects against NAFLD *via* bile-acid-dependent reductions in lipid absorption. Cell. Metab. 33 (8), 1671–1684.e4. 10.1016/j.cmet.2021.06.012 34270928 PMC8353952

[B11] Colonna M. Holtzman D. M. (2025). Rethinking TREM2 as a target for alzheimer's disease after the INVOKE-2 trial failure. Nat. Med. 31, 3217–3218. 10.1038/s41591-025-03816-2 40603729

[B12] Coppe J. P. Desprez P. Y. Krtolica A. Campisi J. (2010). The senescence-associated secretory phenotype: the dark side of tumor suppression. Annu. Rev. Pathol. 5, 99–118. 10.1146/annurev-pathol-121808-102144 20078217 PMC4166495

[B13] de Jesus Simao J. de Sousa Bispo A. F. Plata V. T. G. Abel A. B. M. Telles M. M. Armelin-Correa L. M. (2024). Fish oil attenuates the expression of the CCL2 chemokine and histone-modifying enzymes in LPS-Stimulated human preadipocytes. Metabol. Open 24, 100336. 10.1016/j.metop.2024.100336 39717736 PMC11665696

[B14] Decout A. Katz J. D. Venkatraman S. Ablasser A. (2021). The cGAS-STING pathway as a therapeutic target in inflammatory diseases. Nat. Rev. Immunol. 21 (9), 548–569. 10.1038/s41577-021-00524-z 33833439 PMC8029610

[B15] Deczkowska A. Weiner A. Amit I. (2020). The physiology, pathology, and potential therapeutic applications of the TREM2 signaling pathway. Cell. 181 (6), 1207–1217. 10.1016/j.cell.2020.05.003 32531244

[B16] Endo-Umeda K. Nakashima H. Komine-Aizawa S. Umeda N. Seki S. Makishima M. (2018). Liver X receptors regulate hepatic F4/80 (+) CD11b(+) kupffer cells/macrophages and innate immune responses in mice. Sci. Rep. 8 (1), 9281. 10.1038/s41598-018-27615-7 29915246 PMC6006359

[B17] Escoter-Torres L. Greulich F. Quagliarini F. Wierer M. Uhlenhaut N. H. (2020). Anti-inflammatory functions of the glucocorticoid receptor require DNA binding. Nucleic Acids Res. 48 (15), 8393–8407. 10.1093/nar/gkaa565 32619221 PMC7470971

[B18] Fan J. S. Liu D. N. Huang G. Xu Z. Z. Jia Y. Zhang H. G. (2012). Panax notoginseng saponins attenuate atherosclerosis *via* reciprocal regulation of lipid metabolism and inflammation by inducing liver X receptor alpha expression. J. Ethnopharmacol. 142 (3), 732–738. 10.1016/j.jep.2012.05.053 22683903

[B19] Feng P. Zhu W. Chen N. Li P. He K. Gong J. (2018). Cathepsin B in hepatic kupffer cells regulates activation of TLR4-independent inflammatory pathways in mice with lipopolysaccharide-induced sepsis. Nan Fang. Yi Ke Da Xue Xue Bao 38 (12), 1465–1471. 10.12122/j.issn.1673-4254.2018.12.11 30613015 PMC6744205

[B20] Feng J. Dai W. Mao Y. Wu L. Li J. Chen K. (2020). Simvastatin re-sensitizes hepatocellular carcinoma cells to sorafenib by inhibiting HIF-1α/PPAR-γ/PKM2-mediated glycolysis. J. Exp. Clin. Cancer Res. 39 (1), 24. 10.1186/s13046-020-1528-x 32000827 PMC6993409

[B21] Foretz M. Guigas B. Viollet B. (2023). Metformin: update on mechanisms of action and repurposing potential. Nat. Rev. Endocrinol. 19 (8), 460–476. 10.1038/s41574-023-00833-4 37130947 PMC10153049

[B22] Fu T. Li Y. Oh T. G. Cayabyab F. He N. Tang Q. (2022). FXR mediates ILC-Intrinsic responses to intestinal inflammation. Proc. Natl. Acad. Sci. U. S. A. 119 (51), e2213041119. 10.1073/pnas.2213041119 36508655 PMC9907109

[B23] Fuchs C. D. Sroda N. Scharnagl H. Gupta R. Minto W. Stojakovic T. (2023). Non-steroidal FXR agonist cilofexor improves cholestatic liver injury in the Mdr2(-/-) mouse model of sclerosing cholangitis. JHEP Rep. 5 (11), 100874. 10.1016/j.jhepr.2023.100874 37841639 PMC10568427

[B24] Fyfe J. Dye D. Razak N. B. A. Metharom P. Falasca M. (2023). Immune evasion on the nanoscale: small extracellular vesicles in pancreatic ductal adenocarcinoma immunity. Semin. Cancer Biol. 96, 36–47. 10.1016/j.semcancer.2023.09.004 37748738

[B25] Gao D. Jiao J. Wang Z. Huang X. Ni X. Fang S. (2022). The roles of cell-cell and organ-organ crosstalk in the type 2 diabetes mellitus associated inflammatory microenvironment. Cytokine Growth Factor Rev. 66, 15–25. 10.1016/j.cytogfr.2022.04.002 35459618

[B26] Gao Y. Wang P. Lu S. Ma W. (2023). METTL3 inhibitor STM2457 improves metabolic dysfunction-associated fatty liver disease by regulating mitochondrial function in mice. Nan Fang. Yi Ke Da Xue Xue Bao 43 (10), 1689–1696. 10.12122/j.issn.1673-4254.2023.10.06 37933644 PMC10630206

[B27] Gawrieh S. Noureddin M. Loo N. Mohseni R. Awasty V. Cusi K. (2021). Saroglitazar, a PPAR-α/γ agonist, for treatment of NAFLD: a randomized controlled double-blind phase 2 trial. Hepatology 74 (4), 1809–1824. 10.1002/hep.31843 33811367

[B28] Guan B. Tong J. Hao H. Yang Z. Chen K. Xu H. (2022). Bile acid coordinates microbiota homeostasis and systemic immunometabolism in cardiometabolic diseases. Acta Pharm. Sin. B 12 (5), 2129–2149. 10.1016/j.apsb.2021.12.011 35646540 PMC9136572

[B29] Guo X. Wen S. Wang J. Zeng X. Yu H. Chen Y. (2024). Senolytic combination of dasatinib and quercetin attenuates renal damage in diabetic kidney disease. Phytomedicine 130, 155705. 10.1016/j.phymed.2024.155705 38761776

[B30] Hayward S. L. Wilson C. L. Kidambi S. (2016). Hyaluronic acid-conjugated liposome nanoparticles for targeted delivery to CD44 overexpressing glioblastoma cells. Oncotarget 7 (23), 34158–34171. 10.18632/oncotarget.8926 27120809 PMC5085145

[B31] Hou L. Yang L. Chang N. Zhao X. Zhou X. Dong C. (2020). Macrophage sphingosine 1-Phosphate receptor 2 blockade attenuates liver inflammation and fibrogenesis triggered by NLRP3 inflammasome. Front. Immunol. 11, 1149. 10.3389/fimmu.2020.01149 32695095 PMC7333785

[B32] Hu Y. Yang C. Shen G. Yang S. Cheng X. Cheng F. (2019). Hyperglycemia-triggered Sphingosine-1-Phosphate and Sphingosine-1-Phosphate receptor 3 signaling worsens liver ischemia/reperfusion injury by regulating M1/M2 polarization. Liver Transpl. 25 (7), 1074–1090. 10.1002/lt.25470 30972941 PMC6617772

[B33] Hu X. Zhou J. Song S. S. Kong W. Shi Y. C. Chen L. L. (2020). TLR4/AP-1-Targeted anti-inflammatory intervention attenuates insulin sensitivity and liver steatosis. Mediat. Inflamm. 2020, 2960517. 10.1155/2020/2960517 33013197 PMC7519185

[B34] Ikonomopoulou M. P. Lopez-Mancheño Y. Novelle M. G. Martinez-Uña M. Gangoda L. Pal M. (2021). LXR stimulates a metabolic switch and reveals cholesterol homeostasis as a Statin target in tasmanian devil facial tumor disease. Cell. Rep. 34 (11), 108851. 10.1016/j.celrep.2021.108851 33730574

[B35] Islam M. T. Tuday E. Allen S. Kim J. Trott D. W. Holland W. L. (2023). Senolytic drugs, dasatinib and quercetin, attenuate adipose tissue inflammation, and ameliorate metabolic function in old age. Aging Cell. 22 (2), e13767. 10.1111/acel.13767 36637079 PMC9924942

[B36] Ito A. Hong C. Rong X. Zhu X. Tarling E. J. Hedde P. N. (2015). LXRs link metabolism to inflammation through Abca1-dependent regulation of membrane composition and TLR signaling. Elife 4, e08009. 10.7554/eLife.08009 26173179 PMC4517437

[B37] Jafarzadeh S. Nemati M. Zandvakili R. Jafarzadeh A. (2025). Modulation of M1 and M2 macrophage polarization by metformin: implications for inflammatory diseases and malignant tumors. Int. Immunopharmacol. 151, 114345. 10.1016/j.intimp.2025.114345 40024215

[B38] Jaitin D. A. Adlung L. Thaiss C. A. Weiner A. Li B. Descamps H. (2019). Lipid-associated macrophages control metabolic homeostasis in a Trem2-Dependent manner. Cell. 178 (3), 686–698.e14. 10.1016/j.cell.2019.05.054 31257031 PMC7068689

[B39] Jiang H. He H. Chen Y. Huang W. Cheng J. Ye J. (2017). Identification of a selective and direct NLRP3 inhibitor to treat inflammatory disorders. J. Exp. Med. 214 (11), 3219–3238. 10.1084/jem.20171419 29021150 PMC5679172

[B40] Jung T. W. Lee S. H. Kim H. C. Bang J. S. Abd El-Aty A. M. Hacımüftüoğlu A. (2018). METRNL attenuates lipid-induced inflammation and insulin resistance *via* AMPK or PPARδ-dependent pathways in skeletal muscle of mice. Exp. Mol. Med. 50 (9), 1–11. 10.1038/s12276-018-0147-5 30213948 PMC6137187

[B41] Kanda H. Tateya S. Tamori Y. Kotani K. Hiasa K. i. Kitazawa R. (2006). MCP-1 contributes to macrophage infiltration into adipose tissue, insulin resistance, and hepatic steatosis in obesity. J. Clin. Investig. 116 (6), 1494–1505. 10.1172/JCI26498 16691291 PMC1459069

[B42] Kang J. Liu M. Yang Q. Dang X. Li Q. Wang T. (2025). Exercise training exerts beneficial effects on alzheimer's disease through multiple signaling pathways. Front. Aging Neurosci. 17, 1558078. 10.3389/fnagi.2025.1558078 40469843 PMC12133837

[B43] Kauppinen A. Suuronen T. Ojala J. Kaarniranta K. Salminen A. (2013). Antagonistic crosstalk between NF-κB and SIRT1 in the regulation of inflammation and metabolic disorders. Cell. Signal 25 (10), 1939–1948. 10.1016/j.cellsig.2013.06.007 23770291

[B44] Kheirollahi V. Wasnick R. M. Biasin V. Vazquez-Armendariz A. I. Chu X. Moiseenko A. (2019). Metformin induces lipogenic differentiation in myofibroblasts to reverse lung fibrosis. Nat. Commun. 10 (1), 2987. 10.1038/s41467-019-10839-0 31278260 PMC6611870

[B45] Kirchgessner T. G. Martin R. Sleph P. Grimm D. Liu X. Lupisella J. (2015). Pharmacological characterization of a novel liver X receptor agonist with partial LXRα activity and a favorable window in nonhuman Primates. J. Pharmacol. Exp. Ther. 352 (2), 305–314. 10.1124/jpet.114.219923 25467132

[B46] le Maire A. Grimaldi M. Roecklin D. Dagnino S. Vivat-Hannah V. Balaguer P. (2009). Activation of RXR-PPAR heterodimers by organotin environmental endocrine disruptors. EMBO Rep. 10 (4), 367–373. 10.1038/embor.2009.8 19270714 PMC2672886

[B47] Lee J. H. Gao Z. Ye J. (2013). Regulation of 11beta-HSD1 expression during adipose tissue expansion by hypoxia through different activities of NF-kappaB and HIF-1alpha. Am. J. Physiol. Endocrinol. Metab. 304 (10), E1035–E1041. 10.1152/ajpendo.00029.2013 23512810 PMC3651619

[B48] Lee S. M. Muratalla J. Diaz-Ruiz A. Remon-Ruiz P. McCann M. Liew C. W. (2021). Rosiglitazone requires hepatocyte PPARγ expression to promote steatosis in Male mice with diet-induced obesity. Endocrinology 162 (11), bqab175. 10.1210/endocr/bqab175 34417811 PMC8428295

[B49] Lee E. Ha S. Kim G. Kim J. H. Jin S. M. (2023). Extracellular vesicles derived from three-dimensional-cultured human umbilical cord blood mesenchymal stem cells prevent inflammation and dedifferentiation in pancreatic islets. Stem Cells Int. 2023, 5475212. 10.1155/2023/5475212 36860546 PMC9970714

[B50] Long H. Simmons A. Mayorga A. Burgess B. Nguyen T. Budda B. (2024). Preclinical and first-in-human evaluation of AL002, a novel TREM2 agonistic antibody for alzheimer's disease. Alzheimers Res. Ther. 16 (1), 235. 10.1186/s13195-024-01599-1 39444037 PMC11515656

[B51] Mackinnon A. C. Tonev D. Jacoby B. Pinzani M. Slack R. J. (2023). Galectin-3: therapeutic targeting in liver disease. Expert Opin. Ther. Targets 27 (9), 779–791. 10.1080/14728222.2023.2258280 37705214

[B52] Michelet X. Dyck L. Hogan A. Loftus R. M. Duquette D. Wei K. (2018). Metabolic reprogramming of natural killer cells in obesity limits antitumor responses. Nat. Immunol. 19 (12), 1330–1340. 10.1038/s41590-018-0251-7 30420624

[B53] Milan K. L. Shree R. A. Nandana N. Leela R. Ramkumar K. M. (2025a). Role of macrophages reprogramming in pathogenesis of gestational diabetes mellitus. Cytokine 196, 157041. 10.1016/j.cyto.2025.157041 41033157

[B54] Milan K. L. Nandhana N. Shree R. A. Leela R. Jahnavi S. Anuradha M. (2025b). NEK7-mediated activation of NLRP1 and NLRP3 inflammasomes in the progression of gestational diabetes mellitus. Hum. Immunol. 86 (4), 111331. 10.1016/j.humimm.2025.111331 40435843

[B55] Misra S. Ke C. Srinivasan S. Goyal A. Nyriyenda M. J. Florez J. C. (2023). Current insights and emerging trends in early-onset type 2 diabetes. Lancet Diabetes Endocrinol. 11 (10), 768–782. 10.1016/S2213-8587(23)00225-5 37708901

[B56] Montaigne D. Butruille L. Staels B. (2021). PPAR control of metabolism and cardiovascular functions. Nat. Rev. Cardiol. 18 (12), 809–823. 10.1038/s41569-021-00569-6 34127848

[B57] Motani A. Wang Z. Weiszmann J. McGee L. R. Lee G. Liu Q. (2009). INT131: a selective modulator of PPAR gamma. J. Mol. Biol. 386 (5), 1301–1311. 10.1016/j.jmb.2009.01.025 19452630

[B58] Nahoum V. Pérez E. Germain P. Rodríguez-Barrios F. Manzo F. Kammerer S. (2007). Modulators of the structural dynamics of the retinoid X receptor to reveal receptor function. Proc. Natl. Acad. Sci. U. S. A. 104 (44), 17323–17328. 10.1073/pnas.0705356104 17947383 PMC2077255

[B59] Niu X. Han P. Liu J. Chen Z. Zhang T. Li B. (2023). Regulation of PPARγ/CPT-1 expression ameliorates cochlear hair cell injury by regulating cellular lipid metabolism and oxidative stress. Mol. Genet. Genomics 298 (2), 473–483. 10.1007/s00438-023-01993-8 36639590

[B60] Olona A. Leishman S. Anand P. K. (2022). The NLRP3 inflammasome: regulation by metabolic signals. Trends Immunol. 43 (12), 978–989. 10.1016/j.it.2022.10.003 36371361

[B61] Pan Y. Hui X. Hoo R. L. C. Ye D. Chan C. Y. C. Feng T. (2019). Adipocyte-secreted exosomal microRNA-34a inhibits M2 macrophage polarization to promote obesity-induced adipose inflammation. J. Clin. Investig. 129 (2), 834–849. 10.1172/JCI123069 30667374 PMC6355214

[B62] Pan Y. You Y. Sun L. Sui Q. Liu L. Yuan H. (2021). The STING antagonist H-151 ameliorates psoriasis *via* suppression of STING/NF-kappaB-mediated inflammation. Br. J. Pharmacol. 178 (24), 4907–4922. 10.1111/bph.15673 34460100

[B63] Pan Y. Chen H. Zhang X. Liu W. Ding Y. Huang D. (2023). METTL3 drives NAFLD-Related hepatocellular carcinoma and is a therapeutic target for boosting immunotherapy. Cell. Rep. Med. 4 (8), 101144. 10.1016/j.xcrm.2023.101144 37586322 PMC10439254

[B64] Panzitt K. Wagner M. (2021). FXR in liver physiology: multiple faces to regulate liver metabolism. Biochim. Biophys. Acta Mol. Basis Dis. 1867 (7), 166133. 10.1016/j.bbadis.2021.166133 33771667

[B65] Pasupuleti S. K. Ramdas B. Burns S. S. Palam L. R. Kanumuri R. Kumar R. (2023). Obesity-induced inflammation exacerbates clonal hematopoiesis. J. Clin. Investig. 133 (11), e163968. 10.1172/JCI163968 37071471 PMC10231999

[B66] Patel K. Harrison S. A. Elkhashab M. Trotter J. F. Herring R. Rojter S. E. (2020). Cilofexor, a nonsteroidal FXR agonist, in patients with noncirrhotic NASH: a phase 2 randomized controlled trial. Hepatology 72 (1), 58–71. 10.1002/hep.31205 32115759

[B67] Pepys M. B. Hirschfield G. M. (2003). C-reactive protein: a critical update. J. Clin. Investig. 111 (12), 1805–1812. 10.1172/JCI18921 12813013 PMC161431

[B68] Potì F. Scalera E. Feuerborn R. Fischer J. Arndt L. Varga G. (2024). Sphingosine 1-phosphate receptor 1signaling in macrophages reduces atherosclerosis in LDL receptor-deficient mice. JCI Insight 9 (24), e158127. 10.1172/jci.insight.158127 39531328 PMC11665566

[B69] Prattichizzo F. De Nigris V. Spiga R. Mancuso E. La Sala L. Antonicelli R. (2018). Inflammageing and metaflammation: the yin and yang of type 2 diabetes. Ageing Res. Rev. 41, 1–17. 10.1016/j.arr.2017.10.003 29081381

[B70] Reich T. Adato O. Kofman N. S. Feiglin A. Unger R. (2023). TREM2 has a significant, gender-specific, effect on human obesity. Sci. Rep. 13 (1), 482. 10.1038/s41598-022-27272-x 36627355 PMC9832124

[B71] Ritter M. J. Amano I. Imai N. Soares De Oliveira L. Vella K. R. Hollenberg A. N. (2021). Nuclear receptor CoRepressors, NCOR1 and SMRT, are required for maintaining systemic metabolic homeostasis. Mol. Metab. 53, 101315. 10.1016/j.molmet.2021.101315 34390859 PMC8429965

[B72] Rong S. Cortés V. A. Rashid S. Anderson N. N. McDonald J. G. Liang G. (2017). Expression of SREBP-1c requires SREBP-2-mediated generation of a sterol ligand for LXR in livers of mice. Elife 6, e25015. 10.7554/eLife.25015 28244871 PMC5348127

[B73] Rudalska R. Harbig J. Snaebjornsson M. T. Klotz S. Zwirner S. Taranets L. (2021). LXRα activation and raf inhibition trigger lethal lipotoxicity in liver cancer. Nat. Cancer 2 (2), 201–217. 10.1038/s43018-020-00168-3 35122079

[B74] Sakai M. Troutman T. D. Seidman J. S. Ouyang Z. Spann N. J. Abe Y. (2019). Liver-derived signals sequentially reprogram myeloid enhancers to initiate and maintain kupffer cell identity. Immunity 51 (4), 655–670.e8. 10.1016/j.immuni.2019.09.002 31587991 PMC6800814

[B75] Schlepckow K. Kleinberger G. Fukumori A. Feederle R. Lichtenthaler S. F. Steiner H. (2017). An Alzheimer-associated TREM2 variant occurs at the ADAM cleavage site and affects shedding and phagocytic function. EMBO Mol. Med. 9 (10), 1356–1365. 10.15252/emmm.201707672 28855300 PMC5623859

[B76] Sharma R. Porterfield J. E. An H. T. Jimenez A. S. Lee S. Kannan S. (2021). Rationally designed galactose dendrimer for hepatocyte-specific targeting and intracellular drug delivery for the treatment of liver disorders. Biomacromolecules 22 (8), 3574–3589. 10.1021/acs.biomac.1c00649 34324818

[B77] Tan J. Fan W. Liu T. Zhu B. Liu Y. Wang S. (2023). TREM2(+) macrophages suppress CD8(+) T-cell infiltration after transarterial chemoembolisation in hepatocellular carcinoma. J. Hepatol. 79 (1), 126–140. 10.1016/j.jhep.2023.02.032 36889359

[B78] Tanaka M. Yamakage H. Muranaka K. Yamada T. Araki R. Ogo A. (2022). Higher serum soluble TREM2 as a potential indicative biomarker for cognitive impairment in inadequately controlled type 2 diabetes without obesity: the DOR-KyotoJ-1. Front. Endocrinol. (Lausanne) 13, 880148. 10.3389/fendo.2022.880148 35592778 PMC9110765

[B79] Teodoro J. S. Rolo A. P. Palmeira C. M. (2011). Hepatic FXR: key regulator of whole-body energy metabolism. Trends Endocrinol. Metab. 22 (11), 458–466. 10.1016/j.tem.2011.07.002 21862343

[B80] Wang S. Mustafa M. Yuede C. M. Salazar S. V. Kong P. Long H. (2020). Anti-human TREM2 induces microglia proliferation and reduces pathology in an alzheimer's disease model. J. Exp. Med. 217 (9), e20200785. 10.1084/jem.20200785 32579671 PMC7478730

[B81] Wang Q. Li D. Cao G. Shi Q. Zhu J. Zhang M. (2021). IL-27 signalling promotes adipocyte thermogenesis and energy expenditure. Nature 600 (7888), 314–318. 10.1038/s41586-021-04127-5 34819664

[B82] Wang S. Sudan R. Peng V. Zhou Y. Du S. Yuede C. M. (2022a). TREM2 drives microglia response to amyloid-beta *via* SYK-dependent and -independent pathways. Cell. 185 (22), 4153–4169 e19. 10.1016/j.cell.2022.09.033 36306735 PMC9625082

[B83] Wang J. Q. Li L. L. Hu A. Deng G. Wei J. Li Y. F. (2022b). Inhibition of ASGR1 decreases lipid levels by promoting cholesterol excretion. Nature 608 (7922), 413–420. 10.1038/s41586-022-05006-3 35922515

[B84] Wang X. He Q. Zhou C. Xu Y. Liu D. Fujiwara N. (2023). Prolonged hypernutrition impairs TREM2-dependent efferocytosis to license chronic liver inflammation and NASH development. Immunity 56 (1), 58–77 e11. 10.1016/j.immuni.2022.11.013 36521495 PMC9839616

[B85] Wang K. Zhang Y. Wang G. Hao H. Wang H. (2024). FXR agonists for MASH therapy: lessons and perspectives from obeticholic acid. Med. Res. Rev. 44 (2), 568–586. 10.1002/med.21991 37899676

[B86] Wen C. K. Lee T. Y. (2015). Electroacupuncture prevents white adipose tissue inflammation through modulation of hypoxia-inducible factors-1alpha-dependent pathway in Obese mice. BMC Complement. Altern. Med. 15, 452. 10.1186/s12906-015-0977-9 26714835 PMC4696133

[B87] Wu D. Yan Z. B. Cheng Y. G. Zhong M. W. Liu S. Z. Zhang G. Y. (2018). Deactivation of the NLRP3 inflammasome in infiltrating macrophages by duodenal-jejunal bypass surgery mediates improvement of beta cell function in type 2 diabetes. Metabolism 81, 1–12. 10.1016/j.metabol.2017.10.015 29129820

[B88] Wu X. Fan X. Miyata T. Kim A. Cajigas-Du Ross C. K. Ray S. (2023). Recent advances in understanding of pathogenesis of alcohol-associated liver disease. Annu. Rev. Pathol. 18, 411–438. 10.1146/annurev-pathmechdis-031521-030435 36270295 PMC10060166

[B89] Xie L. Qiu S. Lu C. Gu C. Wang J. Lv J. (2023). Gastric cancer-derived LBP promotes liver metastasis by driving intrahepatic fibrotic pre-metastatic niche formation. J. Exp. Clin. Cancer Res. 42 (1), 258. 10.1186/s13046-023-02833-8 37789385 PMC10546721

[B90] Xu M. Tchkonia T. Ding H. Ogrodnik M. Lubbers E. R. Pirtskhalava T. (2015). JAK inhibition alleviates the cellular senescence-associated secretory phenotype and frailty in old age. Proc. Natl. Acad. Sci. U. S. A. 112 (46), E6301–E6310. 10.1073/pnas.1515386112 26578790 PMC4655580

[B91] Xu Y. Hillman H. Chang M. Barrow F. Ivanov S. Revelo X. S. (2025). Identification of conserved and tissue-restricted transcriptional profiles for lipid associated macrophages. Commun. Biol. 8 (1), 953. 10.1038/s42003-025-08387-z 40550904 PMC12185701

[B92] Yamaguchi J. Tanaka T. Saito H. Nomura S. Aburatani H. Waki H. (2017). Echinomycin inhibits adipogenesis in 3T3-L1 cells in a HIF-Independent manner. Sci. Rep. 7 (1), 6516. 10.1038/s41598-017-06761-4 28747725 PMC5529514

[B93] Yamamoto T. Maurya S. K. Pruzinsky E. Batmanov K. Xiao Y. Sulon S. M. (2023). RIP140 deficiency enhances cardiac fuel metabolism and protects mice from heart failure. J. Clin. Investig. 133 (9), e162309. 10.1172/JCI162309 36927960 PMC10145947

[B94] Yan C. Chen J. Li M. Xuan W. Su D. You H. (2016). A decrease in hepatic microRNA-9 expression impairs gluconeogenesis by targeting FOXO1 in Obese mice. Diabetologia 59 (7), 1524–1532. 10.1007/s00125-016-3932-5 27003684

[B95] Yan C. Zhang Y. Zhang X. Aa J. Wang G. Xie Y. (2018). Curcumin regulates endogenous and exogenous metabolism *via* Nrf2-FXR-LXR pathway in NAFLD mice. Biomed. Pharmacother. 105, 274–281. 10.1016/j.biopha.2018.05.135 29860219

[B96] Yang H. Luo F. Wei Y. Jiao Y. Qian J. Chen S. (2021). TGR5 protects against cholestatic liver disease *via* suppressing the NF-κB pathway and activating the Nrf2/HO-1 pathway. Ann. Transl. Med. 9 (14), 1158. 10.21037/atm-21-2631 34430599 PMC8350648

[B97] Ye X. Song Y. Zhao Y. Zhu D. (2023). Cold stimulation promotes interleukin-4 secretion by mucosal-associated invariant T cells in the adipose tissue to promote adipose browning in mice. Nan Fang. Yi Ke Da Xue Xue Bao 43 (11), 1881–1885. 10.12122/j.issn.1673-4254.2023.11.07 38081605 PMC10713470

[B98] Yee S. W. Chen L. Giacomini K. M. (2012). The role of ATM in response to metformin treatment and activation of AMPK. Nat. Genet. 44 (4), 359–360. 10.1038/ng.2236 22456732 PMC3359140

[B99] Yesian A. R. Chalom M. M. Knudsen N. H. Hyde A. L. Personnaz J. Cho H. (2025). Preadipocyte IL-13/IL-13Rα1 signaling regulates beige adipogenesis through modulation of PPARγ activity. J. Clin. Investig. 135 (11), e169152. 10.1172/JCI169152 40198135 PMC12126228

[B100] Younossi Z. M. Ratziu V. Loomba R. Rinella M. Anstee Q. M. Goodman Z. (2019). Obeticholic acid for the treatment of non-alcoholic steatohepatitis: interim analysis from a multicentre, randomised, placebo-controlled phase 3 trial. Lancet 394 (10215), 2184–2196. 10.1016/S0140-6736(19)33041-7 31813633

[B101] Yue X. Izcue A. Borggrefe T. (2011). Essential role of mediator subunit Med1 in invariant natural killer T-cell development. Proc. Natl. Acad. Sci. U. S. A. 108 (41), 17105–17110. 10.1073/pnas.1109095108 21949387 PMC3193226

[B102] Zhang Y. Li Z. Liu X. Chen X. Zhang S. Chen Y. (2023). 3-Hydroxybutyrate ameliorates insulin resistance by inhibiting PPARγ Ser273 phosphorylation in type 2 diabetic mice. Signal Transduct. Target Ther. 8 (1), 190. 10.1038/s41392-023-01415-6 37230992 PMC10212965

[B103] Zhang X. McDonald J. G. Aryal B. Canfrán-Duque A. Goldberg E. L. Araldi E. (2021). Desmosterol suppresses macrophage inflammasome activation and protects against vascular inflammation and atherosclerosis. Proc. Natl. Acad. Sci. U. S. A. 118 (47), e2107682118. 10.1073/pnas.2107682118 34782454 PMC8617522

[B104] Zhang Y. Lv J. Bai J. Zhang X. Wu G. Lei X. (2024). METTL3 modulates TXNIP expression to affect the activation of NLRP3 inflammasome in hepatic cells under oxygen-glucose deprivation/reperfusion injury. Inflammation 47 (3), 1028–1040. 10.1007/s10753-023-01958-4 38236385

[B105] Zhang L. Xiang X. Li Y. Bu G. Chen X. F. (2025). TREM2 and sTREM2 in alzheimer's disease: from mechanisms to therapies. Mol. Neurodegener. 20 (1), 43. 10.1186/s13024-025-00834-z 40247363 PMC12004684

[B106] Zheng H. Jia L. Liu C. C. Rong Z. Zhong L. Yang L. (2017). TREM2 promotes microglial survival by activating Wnt/β-Catenin pathway. J. Neurosci. 37 (7), 1772–1784. 10.1523/JNEUROSCI.2459-16.2017 28077724 PMC5320608

[B107] Zheng Y. Zhang J. Zhao Y. Zhang Y. Zhang X. Guan J. (2021). Curcumin protects against cognitive impairments in a rat model of chronic cerebral hypoperfusion combined with diabetes mellitus by suppressing neuroinflammation, apoptosis, and pyroptosis. Int. Immunopharmacol. 93, 107422. 10.1016/j.intimp.2021.107422 33548579

[B108] Zheng S. Que X. Wang S. Zhou Q. Xing X. Chen L. (2023). ZDHHC5-mediated NLRP3 palmitoylation promotes NLRP3-NEK7 interaction and inflammasome activation. Mol. Cell. 83 (24), 4570–4585 e7. 10.1016/j.molcel.2023.11.015 38092000

[B109] Zheng W. Zhang Y. Xu P. Wang Z. Shao X. Chen C. (2024). TFEB safeguards trophoblast syncytialization in humans and mice. Proc. Natl. Acad. Sci. U. S. A. 121 (28), e2404062121. 10.1073/pnas.2404062121 38968109 PMC11253012

[B110] Zhong J. Xing X. Gao Y. Pei L. Lu C. Sun H. (2024). Distinct roles of TREM2 in central nervous system cancers and peripheral cancers. Cancer Cell. 42 (6), 968–984.e9. 10.1016/j.ccell.2024.05.001 38788719

[B111] Zhou T. Xu X. Du M. Zhao T. Wang J. (2018). A preclinical overview of metformin for the treatment of type 2 diabetes. Biomed. Pharmacother. 106, 1227–1235. 10.1016/j.biopha.2018.07.085 30119191

[B112] Zhou W. Zhang X. R. Qin X. C. Xie Y. C. Zhang D. Wang Z. C. (2025). Naringin mitigates experimental autoimmune prostatitis by modulating oxidative stress and the NLRP3 inflammasome *via* the PPAR-γ/NF-κB pathway. Sci. Rep. 15 (1), 20843. 10.1038/s41598-025-04916-2 40594232 PMC12214577

[B113] Zou G. Tang Y. Yang J. Fu S. Li Y. Ren X. (2025). Signal-induced NLRP3 phase separation initiates inflammasome activation. Cell. Res. 35 (6), 437–452. 10.1038/s41422-025-01096-6 40164768 PMC12134225

